# Impact of age and level of experience on occupational stress experienced by non-gazetted officers of the central reserve police force

**DOI:** 10.4103/0972-6748.62264

**Published:** 2009

**Authors:** C. Balakrishnamurthy, Swetha Shankar

**Affiliations:** Department of Psychology, PSG College of Arts and Science, Coimbatore, India

**Keywords:** Occupational stress, Police personnal, Stress

## Abstract

**Background:**

The study explores the effect of demographic variables such as age and level of experience on the level of stress experienced by non-gazette officers of the Central Reserve Police Force (CRPF).

**Materials and Methods:**

A purposive sample of 163 CRPF personnel was chosen. The Police Stress Inventory developed for use among CRPF personnel was administered. Various statistical parameters such as mean, standard deviation, standard error, mean difference and single-factor ANOVA were used to analyze the data.

**Conclusion:**

The study strongly indicates the relationship between stress and demographic variables such as age and level of experience.

As stated by Fred Luthans (2007), the phenomenon of stress has been examined in several disciplines, including psychology, anthropology, physiology, medicine and management. However, the concept of stress was first proposed by Hans Selye (1936). Selye’s well-known definition of stress, based on his research, is that stress is “the nonspecific response of the body to any demand made upon it” (Selye, 1974).

Zeidner and Endler (1996) defined the term “stress” as a condition that arises when an individual experiences a demand that exceeds his or her real or perceived abilities to successfully cope with it, resulting in disturbance to his or her physiological and psychological equilibrium.

In our complex modern society, there has been an increased level of stress, especially for police personnel. This has triggered many problems, in both personal and professional life. In today’s fast-paced world, police personnel are experiencing more stress at every stage of their lives than ever before.

Juggling job pressures, family schedules, money issues, career, educational advancement, concerns about children’s and elders’ care are only a few of the common stressful aspects confronting the personnel. Consequently, adjustment at home and in the workplace is very hazardous to them. Invariably, all the cadres do face many problems such as personal, familial and professional and other social problems, which put great pressure on their commitments. The work status, multiple roles in the family, their psychological and demographic makeup, etc., may drag them to be stress prone. However, the extent to which the independent and combined effects of these variables influence the stress levels makes an interesting scientific problem.

Psychological and sociological factors influence health and physical problems in two distinct ways. First, they can affect the basic biological processes that lead to illness and disease. Second, longstanding behavior patterns may put people at risk to develop certain physical disorders. Sometimes, both these avenues contribute to the etiology or maintenance of disease.

Reddy and Ramamurthy (1997) concluded that stress was found to be considerably influenced by age-related factors among 200 male executives.

Sharma (2007) reported a finding from a study by the Defence Institute of Psychological Research (DIPR) which revealed that increase in the occupational factors such as years of job experience and job hierarchy increased the levels of stress among officers, junior commissioned officers (JCO) and *jawans*.

## Objective

The present study attempts to determine the relationship between demographic variables and levels of stress among CRPF personnel.

## MATERIALS AND METHODS

### The population and sample used in the study

The present study was conducted at the Central Training College-II, Coimbatore.

#### Population

Totally, 2000 police personnel were undergoing training at the Central Training College-II during the study period. From the above source, the desirable sample for the study was arrived at by adapting the purposive sampling technique.

#### Sample selection

The population of the study was restricted to male CRPF personnel with a minimum of 7 years of job experience. Finally, 163 personnel were selected for this study.

### Tool used

Police Personnel Stress Inventory (2007) was used to determine the stress score of the CRPF personnel. It consists of 38 items. Each item of the scale was prepared after intense consultation with the seasoned senior personnel irrespective of rank. The reliability index was ascertained on 40 subjects, using spilt-half (odd-even) method. The correlation coefficient adapted for this scale was found to be .87. The validity of the Police Personnel Stress Inventory was determined by computing coefficient of correlation, which was found to be .62 (*n* = 40).

### Variables

#### Independent variables

Age and level of experience of the personnel.

#### Dependent variable

The stress score obtained from the Police Personnel Stress Inventory.

### Data analysis

Descriptive and inferential statistical parameters such as percentage mean, standard deviation (SD), mean difference (MD), standard error (SE), Student *t test*, critical ratio, single-factor analysis of variance, and Pearson product moment correlation coefficient were appropriately used in order to test the hypothesis.

## RESULTS AND DISCUSSION

Table 1 shows the breakup of ages of the personnel. The personnel were categorized into two major age groups, namely, 27-36 years and 37-57 years. The mean age of personnel falling in the first group (range, 27-36 years) was 34.67 years, whereas the mean age of the elder group of personnel (range, 37-57 years) was 44.68 years. The exact difference between the mean ages of the above groups is 10.01 years.

**Table 1:** Distribution of central reserve police force personnel by age (*n* = 163)

Age levels (years)	*n*	Mean	SD
27-36	75	34.67	1.77
37-57	88	44.68	8.02

Table 2 shows the comparison of stress scores of CRPF personnel by age levels. The stress levels of both the groups of CRPF personnel did not differ significantly, indicating that stress levels were not affected significantly by age. Whereas the mean stress score of personnel in the age group 27-36 years shows they had slightly higher amount of stress than their immediate elders. The actual mean difference was 2.73. Mostofsky and Barlow (2000) and Taylor *et al*. (2000) gave evidence that the same kinds of causal factors active in psychological disorders — social, psychological and biological — play a role in some of the physical disorders; however, the factor attracting the greatest attention is stress, particularly the neurobiological components of the stress response.

**Table 2:** Comparison of stress scores of central reserve police force personnel by age levels

Age levels (*n* = 163)	Stress scores		Mean diff.	SE	CR
	<27 years (*n* = 75)	>37 years (*n* = 88)			
Mean	118.33	115.60	2.73	2.59	1.05
SD	16.45	16.58			

Stress physiology is profoundly influenced by psychological and social factors (Kemeny, 2003).

Table 3 shows stress levels of CRPF personnel classified in terms of 4 major occupational experience categories. As compared to all the four groups of stress level, the arrived F value (3.88) was greater than the table value at 0.01 level. It indicates apparent and significant differences exist between the CRPF personnel’s stress levels and their distinct occupational experiences. Among the above, the experience group, viz., with experience between 11 and 20 years, had markedly higher amount of stress than their counterparts, including the entire group taken together. Interestingly, the mean stress score of the group with experience of 21 to 30 years revealed low levels of stress when compared with all the other experience groups. This means more experienced people had learnt certain stress-coping tactics in the course of their experience, thereby enabling them to effectively deal with the stress triggered due to their personal and professional commitments. This trend was inverted in the less experienced groups.

**Table 3:** Comparison and relationship between experience and stress levels of central reserve police force personnel

Groups (years)	*n*	Stress		Experience		r	F
		Mean	SD	Mean	SD		
<10	9	113.00	12.54	8.33	2.06	−0.16	3.88**
11-20	115	119.58	16.22	13.10	1.12	−0.08	
21-30	8	107.63	19.02	27.25	2.66	−0.02	
>31	31	110.26	15.74	35.68	1.11	−0.02	
Overall	163	116.86	16.53	17.83	9.34	−0.23**	

F = **P < .01 = 2.66; r = **P < .01 = 0.208

A research finding has been repeatedly confirmed by Katon (2003), viz., those who developed psychological disorders were chronically ill or died at a significantly higher rate than men who remained well adjusted and free from psychological disorders.

Cohen and Herbert (1996) illustrated pathways through which psychological and social factors may influence immune system functioning. Direct connections between the brain [central nervous system (CNS)], HYPAC axis (hormonal) and the immune system have already been described. Behavioral changes in response to stressful events, such as increased smoking or poor eating habits, may also suppress the immune system.

When the correlation coefficients between stress and levels of experience are compared, individuals with greater experience exhibit lower levels of stress than those with fewer years of experience. When overall stress scores were considered, experience was found to have a significant impact on job stress of the personnel (–0.23 at 0.01 level). Hence the null hypothesis (1) is accepted.

## CONCLUSION

In the present study, an attempt was made to examine the relationship between stress levels and demographic variables of nongazetted officers of the Central Reserve Police Force. High stress results in poor performance towards predisposing factors such as personal and professional commitments.

Therefore, it can be concluded that demographic variables such as age and level of experience significantly impact the level of stress experienced by CRPF personnel.

### Implication

The understanding gained from the present study is expected to be useful in planning welfare programs for CRPF personnel. The information obtained may provide guidelines to prepare suitable or tailor-made stress-management strategies and to launch programs to overcome stress and enhance the well-being of personnel.
